# TSC/mTOR Pathway Mutation Associated Eosinophilic/Oncocytic Renal Neoplasms: A Heterogeneous Group of Tumors with Distinct Morphology, Immunohistochemical Profile, and Similar Genetic Background

**DOI:** 10.3390/biomedicines10020322

**Published:** 2022-01-29

**Authors:** Kristyna Pivovarcikova, Reza Alaghehbandan, Tomas Vanecek, Riuko Ohashi, Tomas Pitra, Ondrej Hes

**Affiliations:** 1 Department of Pathology, Faculty of Medicine in Pilsen, University Hospital Pilsen, Charles University in Prague, 30460 Pilsen, Czech Republic; vanecek@biopticka.cz (T.V.); hes@biopticka.cz (O.H.); 2 Department of Pathology, Faculty of Medicine, University of British Columbia, Royal Columbian Hospital, Vancouver, BC V3L 3W7, Canada; reza.alaghehbandan@fraserhealth.ca; 3 Histopathology Core Facility, Niigata University Faculty of Medicine, 1-757 Asahimachi-dori, Chuo-ku, Niigata 951-8510, Japan; riuko@med.niigata-u.ac.jp; 4 Department of Urology, Faculty of Medicine in Pilsen, University Hospital Pilsen, Charles University in Prague, 30599 Pilsen, Czech Republic; pitrat@fnplzen.cz

**Keywords:** kidney, oncocytic, eosinophilic, mTOR, chromophobe, renal, tumor, LOT, EVT, ESC

## Abstract

A number of recently described renal tumor entities share an eosinophilic/oncocytic morphology, somewhat solid architectural growth pattern, and tendency to present as low-stage tumors. The vast majority of such tumors follow a non-aggressive clinical behavior. In this review, we discuss the morphological, immunohistochemical, and molecular genetic profiles of the three most recent novel/emerging renal entities associated with TSC/mTOR pathway mutations. These are eosinophilic solid and cystic renal cell carcinoma, eosinophilic vacuolated tumors, and low-grade oncocytic tumors, which belong to a heterogeneous group of renal tumors, demonstrating mostly solid architecture, eosinophilic/oncocytic cytoplasm, and overlapping morphological and immunohistochemical features between renal oncocytoma and chromophobe renal cell carcinoma. All three tumors also share a molecular genetic background with mutations in the mTORC1 pathway (*TSC1/TSC2/mTOR/RHEB*). Despite the common genetic background, it appears that the tumors with *TSC/mTOR* mutations represent a diverse group of distinct renal neoplasms.

## 1. Introduction

Tuberous sclerosis complex (TSC) proteins are important players in regulating the activity of the mammalian target of rapamycin complex 1 (mTORC1). Germline loss-of-function mutation in one of the *TSC* genes leads to the development of a multisystem disorder with phenotypic heterogeneity associated with TSC. TSC syndrome is an autosomal dominant inherited disease manifesting with a wide spectrum of symptoms, including neurological involvement, hamartomas, and tumors in multiple organs. However, two-thirds of TSC patients have de novo germline mutation [1]. The clinical manifestation in TSC (severity and the spectrum of organs involved) can vary widely between patients (even between individuals of the same family carrying the same germline mutation) [2]. In these patients, renal involvement is of high importance because it is prevalent in 55 to 75% of the patients with TSC [3,4,5,6], significantly affecting the patients (both morbidity and mortality). The most common renal tumor seen in TSC patients is angiomyolipoma (AML), while, less frequently, so-called TSC-associated renal cell carcinoma (TSC-RCC) has also been reported. TSC-RCC may have various histologic features as it has been reported in the literature [7,8].

Concurrently, the current literature showed the presence of mTORC1 pathway alteration (*TSC1/TSC2/mTOR/RHEB*) in a number of recently described renal tumors [9] in patients without *TSC* mutations (i.e., in sporadic tumors in patients without germinal mutation of *TSC1* or *TSC2*). These sporadic renal tumors associated with TSC/mTOR pathway alteration appear to form a heterogeneous group of tumors with distinct morphological features and immunohistochemical profiles but different biological behaviors.

The detailed analysis of previously described TSC-RCC showed morphological and immunohistochemical overlap with the mTORC1 pathway altered sporadic renal tumors. The presence of essentially identical renal tumors occurring in the setting of both the germline loss-of-function mutation of *TSC* in patients with TSC and in those without TSC suggests the existence of a sporadic and hereditary counterpart in these tumors.

## 2. Eosinophilic Solid and Cystic-Renal Cell Carcinoma (ESC-RCC)

In 2016, Trpkov et al. described 16 sporadic cases of ESC-RCC, demonstrating distinct morphological, immunohistochemical, and molecular-genetic features [10]. A great majority of ESC-RCCs are sporadic and occur in non-syndromic settings, while a subset of identical tumors has been documented in patients with TSC. Following the study of Trpkov et al., a number of papers from various institutions were published, further supporting the concept of ESC-RCC as a novel entity [11,12,13,14]. Most of the studies reported in the literature are focused on sporadic cases [7,15]. Sporadic ESC-RCC usually affects middle-age or older women and is typically associated with indolent behavior [10,11]. So far, there have been no reports of cancer-specific deaths due to ESC-RCC. However, considering rare cases of ESC-RCC that have been documented with metastatic disease justifies the “RCC” designation for this entity and further clinical follow-up and surveillance in these patients [13,14,16,17,18]. We believe that cases of ESC-RCC were likely previously diagnosed as unclassified RCC or unclassified renal neoplasm (or RCC) with oncocytic/eosinophilic morphology. The true incidence of ESC-RCC is currently unknown. ESC-RCCs are typically smaller, solitary, and usually low-stage tumors. However, multifocal and bilateral tumors have been described in the literature [19].

**Figure 1:**
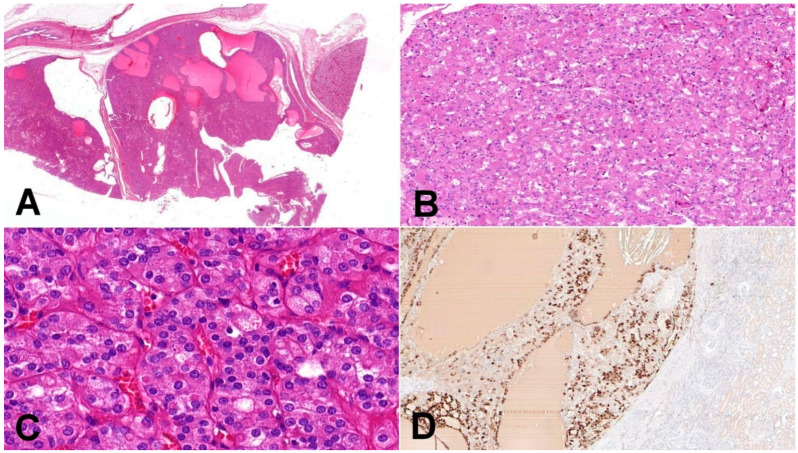
Eosinophilic solid and cystic renal cell carcinoma (ESC-RCC). (**A**) Tumors are solid and cystic, composed of dense population of eosinophilic cells (20×); (**B**) cell population is composed of larger eosinophilic cells (as a predominant cell type) and minor component with cells showing paler cytoplasm (100×); (**C**) in high magnification, cytoplasmic stippling is characteristic but non-specific feature of ESC-RCC (200×); (**D**) CK20 immunohistochemistry—more than 80% of ESC-RCCs are positive for CK20 (200×).

The nomenclature proposed for the entity includes “solid and cystic”, which perfectly conveys and represents the typical macroscopic features of this tumor. ESC-RCC is a well-circumscribed tumor without a fibrous capsule. Microscopically, ESC-RCC is usually arranged in a combination of solid areas (showing diffuse and compact acinar or nested growth patterns) and variably sized macrocystic and microcystic spaces (Figure 1). The cystic spaces are lined by neoplastic cells with a pronounced hobnail arrangement. The tumor cells show an abundant eosinophilic cytoplasm with prominent granular cytoplasmic stippling [10]. The presence of easily identifiable coarse cytoplasmic granules (reminiscing “leishmania bodies”, representing the aggregates of a rough endoplasmic reticulum) [10], is a helpful morphological feature [20]. The nuclei are round to oval, but the nucleoli are generally not prominent. Clusters of admixed foamy histiocytes and lymphocytes are frequently present. Rarely, foci with papillary structures can be found. Very rarely, clusters of “clear/pale cell” or clusters of multinucleated cells might be present in some cases. Psammoma bodies or microcalcifications are found in some cases [18]. An interesting case of ESC-RCC with melanin production was published recently [21].

ESC-RCCs have predominant diffuse or focal CK20 positivity in the majority of cases, and tumors are usually CK7 negative or only focally positive. This immunohistochemical profile is distinct and helpful for routine diagnostic work-up. It should be noted that negative CK20 can be observed in 10 to 15% of otherwise typical ESC-RCC cases with either negative or focally positive CK7 [10,11]. ESC-RCCs are usually positive for PAX8, AE1/3, and vimentin, while being negative for CD117, HMB45, and Melan A [18]. Cathepsin K is also positive in the majority of ESC-RCC [15], either diffuse or focal.

ESC-RCC harbors *TSC1* or *TSC2* mutations; recurrent mutually exclusive somatic bi-allelic loss of *TSC1/2* is considered as a molecular marker of ESC-RCC [13,22,23]. A lack of germline *TSC* aberration in matched non-neoplastic renal parenchyma distinguishes ESC-RCC from its syndromic counterpart (TSC-RCC) [22].

## 3. Low-Grade Oncocytic Tumor (LOT)

LOT was first described in 2019 in a cohort of 28 patients [24]. In the initial study by Trpkov et al., LOT was characterized by a typical CD117 negative/CK7 positive immunoprofile and consistent distinct morphology. Subsequently, multiple studies have been conducted across the global pathology community, identifying to date more than 100 LOT cases that were previously classified as renal oncocytoma [25,26], eosinophilic variant of chromophobe RCC [25,27], unclassified RCC, or “low-grade eosinophilic renal neoplasm” [25,28]. Morini et al. reported the incidence of LOT to be 3.6% of all chromophobe RCCs [27]. The incidence of LOT in tumors previously misclassified as renal oncocytoma is reported at 4.18% by Kravtsov et al. [26]. Based on the current knowledge, LOT seems to be a new distinct entity that can present in both syndromic and non-syndromic settings (tumors with the same morphology were described in patients with TSC [25]). Most recently, LOT was proposed to be considered as a provisional entity by the Genitourinary Pathology Society (GUPS) [18].

LOT is typically a solitary, small tumor that shows low stage, and it is associated with indolent clinical behavior based on the available data. Recent studies have shown that LOT may rarely be multiple and/or bilateral [17,25,29]. LOT is typically a tan/brown, solid tumor, which may show edematous or hemorrhagic focal areas on a cut surface [24,27,28,30,31].

**Figure 2:**
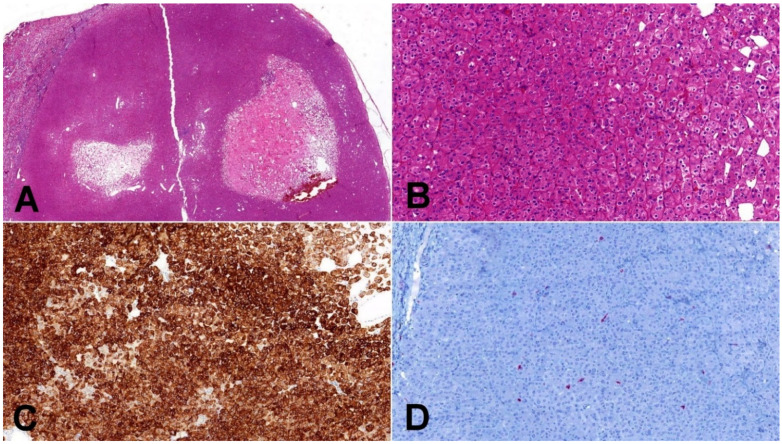
Low-grade oncocytic tumor (LOT). (**A**) Typical LOT presents as small, well-demarcated tumor with areas of regressive changes (20×); (**B**) cells have distinct cytoplasmic borders, round slightly irregular nuclei, and delicate perinuclear clearing (100×); (**C**) CK7 immunohistochemistry—LOT express diffusely CK7 (100×); (**D**) CD117 immunohistochemistry—characteristic immunohistochemical feature of LOT is negative staining for CD117 (internal positive control is clearly visible in present macrophages) (100×).

Histologically, LOT is a noncapsulated tumor with solid, compact nested, or focal tubular growth (notably in the central parts) (Figure 2). The tumor frequently has sharply delineated loose stromal and edematous areas. These areas contain cells typically elongated in shape (sometimes described as of myoid shape) [18,24] arranged in loose reticular, cord-like, and individual single-cell growth [18]. Entrapped non-neoplastic tubules may occasionally be present on the periphery of the tumorous mass. The neoplastic cells have a homogeneous oncocytic cytoplasm with round to oval nuclei, and smooth nuclear membrane (lack significant irregularities and “raisinoid shape”). Delicate perinuclear halos/clearing may be focally present in the nuclei.

LOTs consistently show diffuse strong CK7 positivity and CD117 negativity (CK7+/CD117−), which is considered as a key diagnostic immunohistochemical feature [24]. Such a distinct immunoprofile separates LOTs from other neoplastic mimickers with eosinophilic/oncocytic cytoplasm (i.e., renal oncocytoma and chromophobe RCC, eosinophilic variant). It should be noted that rare cases of LOTs may show CD117 reactivity [17,29]; however, positivity is focal and weak.

In CGH analysis, recurrent deletions of 19p33.3, 1p36.33, and 19q13.11 were found, but even the disomic pattern has been described [24]. Recent studies highlighted the important role of the mTOR pathway in the tumorigenesis of LOT, mostly due to activating *MTOR* mutation is most cases, rarely due to *TSC1* inactivating mutation [27]. Further, mTORC1 activation is probably a typical event in LOT, and this unique molecular background distinguishes these tumors from renal oncocytoma and chromophobe renal cell carcinoma [25].

## 4. Eosinophilic Vacuolated Tumor (EVT)

EVT is a new consensual name proposed by the Genitourinary Pathology Society (GUPS) for a distinct oncocytic tumor described under different names in the literature [18]. EVT was first described in two initial studies as “high-grade oncocytic tumor/HOT” [32] and “sporadic RCC with eosinophilic and vacuolated cytoplasm” [33]. Similar to LOT, EVT was initially considered as a sporadic tumor, but, in subsequent studies, an identical tumor was described in TSC patients [9,25,34]. Based on the limited clinical data, EVT seems to follow a non-aggressive behavior [18], with no disease recurrence or progression during relative long follow-up ranging from 12 to 198 months (mean 56.3, median 41.5 months) [35].

**Figure 3:**
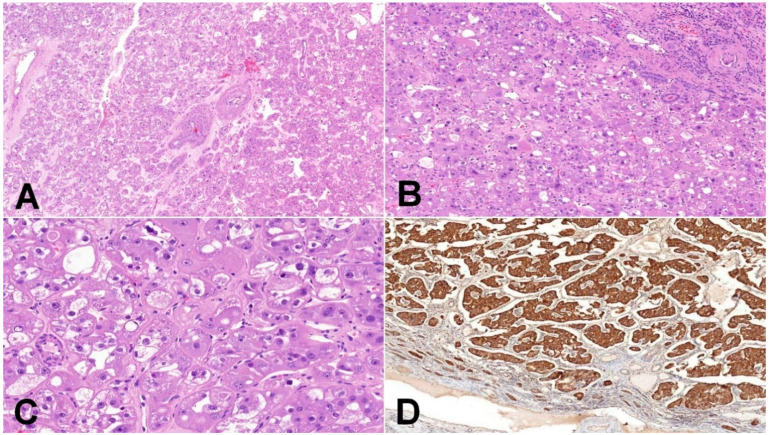
Eosinophilic vacuolated tumor (EVT). (**A**) EVT is composed of oncocytic cells and contains frequently thick-walled vessels (50×); (**B**) EVT is well-demarcated tumor, and the transition between non-neoplastic kidney parenchyma and tumor is usually sharp, with frequent entrapped non-neoplastic tubules (100×); (**C**) neoplastic cells have distinct cytoplasmic membranes, voluminous intracytoplasmic vacuoles, and nuclei with prominent nucleoli (equivalent of grade 3 ISUP/WHO) (200×); (**D**) mitochondrial antigen antibody (MIA) immunohistochemistry—oncocytic characteristic of neoplastic cells can be demonstrated using mitochondrial antigen antibody (100×).

These tumors more frequently occur in women (M:F = 1:2.5) and are found in patients with a broad age range from 25 to 73 years (mean 50.9, median 54 years) [18,32,33]. EVTs are usually low-stage solitary tumors. On cross section, they are described as solid, tan to brown, and without necrotic or hemorrhagic areas [18,32,33,35]. EVT has a readily identifiable morphology that does not fit any of the previously described renal entities. The tumors are arranged in solid to nested architecture, focally with tubulocystic areas. Typically, they are composed of large eosinophilic cells with voluminous intracytoplasmic vacuoles, prominent cell membranes, and oval nuclei with enlarged nucleoli (corresponding to nuclear grade WHO/ISUP 3—Figure 3) [36]. The constant presence of large nuclei with prominent nucleoli leads the authors of the initial study to describe EVT under the name “high-grade oncocytic tumor” [32] despite its obviously indolent behavior. Thick-walled vessels and entrapped tubules are also a common finding [32].

The tumors are consistently positive for CD117, CD10, antimitochondrial antigen antibody, and cathepsin K. Further, the majority of these tumors are positive for PAX8, AE1/AE3, and CK18 [9,32,35]. CK7 is negative or restricted to rare scattered cells [33,35].

He et al. found a loss of chromosome 1, chromosome 19, loss of heterozygosity at 16p11, and 7q31 [32]. The loss of chromosome 1 concurrently with activating *MTOR* mutations was also documented by others [9,33]. The association of EVT and mTOR pathway abnormalities, including non-overlapping mutations in *mTOR*, *TSC2*, and *TSC1*, was confirmed in a recent multi-institutional study [35]. In one case described by Farcas et al., an *mTOR* mutation showed a coexistence of *RICTOR* missense mutation [35]. Thus, EVT appears to develop in the setting of germline or somatic inactivating mutations, leading to mTORC1 activation [9].

Molecular karyotype analyses showed partly overlapping chromosomal abnormalities, both in EVT as well as in LOT. These include, for example, the loss of chromosome 1, where many genes involved in carcinogenesis are localized, including *mTOR*. Another common change involves chromosome 19, containing mTOR pathway genes *MAP2K2*, *EEF,* or *AKT1S1*. What the exact role of these changes is in the pathogenesis of EVT or LOT remains to be elucidated by future studies.

## 5. Differential Diagnosis

**Figure 4:**
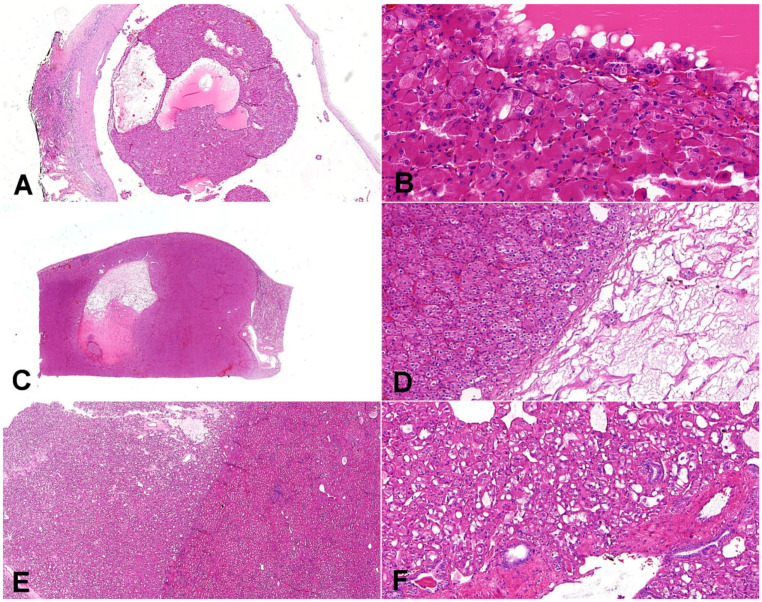
Composite comparative figure of discussed entities. (**A**) Eosinophilic solid and cystic renal cell carcinoma (ESC-RCC) typically combines the solid and cystic areas (20×); (**B**) ESC-RCC is composed of cells with abundant eosinophilic cytoplasm with prominent granular cytoplasmic stippling (“leishmania bodies”) (200×). (**C**) Low-grade oncocytic tumor (LOT) is typically well-demarcated (10×); (**D**) LOT has frequent sharply delineated loose stromal and edematous areas (100×). (**E**) Eosinophilic vacuolated tumor (EVT) is well-demarcated tumor (20×); (**F**) the neoplastic cells are voluminous, eosinophilic, with prominent cell membranes and intracytoplasmic vacuoles; on the periphery, there are entrapped non-neoplastic tubules frequently (100×).

All three novel/emerging renal tumors (ESC-RCC, LOT, and EVT) demonstrate unique morphology (Figure 4), relatively consistent immunoprofiles, and distinct molecular genetic features. ESC-RCC is an eosinophilic tumor, which is not oncocytic sensu stricto. The cytoplasm in ESC-RCC is filled by a broad spectrum of organelles, and mitochondria are not predominant [10]. EVT and LOT are true oncocytic tumors (composed of epithelial cells stuffed with mitochondria). These tumors emerged from the spectrum of “hybrid oncocytic–chromophobe renal tumors“, or from the so-called “unclassified eosinophilic tumors”. None of the above-mentioned tumors fit into any of the traditionally or currently recognized renal tumor categories, such as renal oncocytoma or chromophobe RCC [37,38]. All three tumors have been proposed as novel or emerging new entities by the GUPS [18].

The broad spectrum of the renal tumors with oncocytic/eosinophilic cells should be considered in the differential diagnosis of ESC-RCC, LOT, and EVT. This would include renal oncocytoma, eosinophilic variant of chromophobe RCC, succinate dehydrogenase (SDH)-deficient RCC, MiTF translocation RCC (particularly *TFEB*), and epithelioid angiomyolipoma (AML). A summary of the features of these entities, useful in the differential diagnosis, is given in Table 1.

The morphological features of ESC-RCC observed on H&E and its immunohistochemical profile are generally sufficient for the diagnosis. ESC-RCC presents with its typical histological features of solid and cystic architecture and neoplastic cells with voluminous eosinophilic cytoplasm, but not oncocytic. The cytoplasm lacks predominant mitochondria as a main organelle, resulting in patchy (not diffuse) MIA (mitochondrial antigen antibody) positivity. The nuclei may be irregular, but they lack a raisinoid shape and perinuclear clearing, which is typical for chromophobe RCC. Frequent CK20 positivity aids in distinguishing ESC-RCC from chromophobe RCC. Vimentin may be another marker helping in the differential diagnosis between ESC-RCC and chromophobe RCC or renal oncocytoma. Vimentin is usually negative in chromophobe RCC and negative or only focally positive in renal oncocytoma (positivity of single cells, usually near to central scary area) [38]. ESC-RCC is typically vimentin positive.

ESC-RCC also lacks neoplastic cell uniformity, which is typically seen in LOT. Further, the lack of central edematous or hemorrhagic tumor areas with typical elongated cells in ESC-RCC is also worth noting (these features are typically seen in LOT). In limited material, the immunohistochemical profile (CK7, CK20, CD117) can assist in rendering an accurate diagnosis.

Gupta et al. [39] recently reported a case of CK7+/CD117− oncocytic neoplasm, immunophenotypically compatible with LOT but showing significant nuclear membrane irregularity, perinuclear halos, and occasional binucleation. Further, the tumor did not show any chromosomal copy number changes. For such cases with overlapping features with chromophobe RCC, classification and prognostic significance currently remain uncertain.

EVT has a typical “high-grade” appearance with voluminous cytoplasm, distinct cytoplasmic membranes, and prominent intracytoplasmic vacuoles. EVT is easily distinguishable from ESC-RCC on a histologic basis. However, in some rare cases of ESC-RCC, occasional intracytoplasmic vacuoles may focally be present (such cells do not form the main neoplastic population). Thick walled-vessels, which are seen in EVT, are not typically present in ESC-RCC, and entrapped non-neoplastic tubules are also very rarely seen in ESC-RCC. The immunohistochemical profile might be helpful, but it is important to note that ESC-RCC can rarely be CK20 negative, while EVT can rarely be CK20 positive in single cells. Classic chromophobe RCC with dual cell populations of voluminous pale cells and smaller pink cells can potentially resemble EVT. However, chromophobe RCC lacks marked cytoplasmic vacuoles, “atypical” nuclear features with very prominent nucleoli, while exhibiting irregular (“raisinoid”) nuclei (which is not observed in EVT). However, chromophobe RCC with variant morphologies (i.e., pigmented, adenomatoid) might mimic EVT (but large intracytoplasmic vacuoles are typically missing in such tumors). Both oncocytoma and chromophobe RCC demonstrate CD117 reactivity and are negative for cathepsin K, which is in opposition to the EVT and ESC-RCC immunoprofiles. Chromophobe RCC also typically shows diffuse CK7 reactivity, unlike EVT and ESC-RCC. Besides classic cases with diffuse strong CK7 positivity, chromophobe RCC can express CK7 in variable intensity, with clusters of cells staining a strongly membranous aspect [40]. However, this pattern is different from ESC-RCC and EVT. Farcas et al. recently presented an important differential diagnostic dilemma. In their series, two tumors, which were nearly identical to EVT, showed mutations in the *folliculin (BHD)* gene. These tumors shared identical cytologic and immunohistochemical features with EVT; however, the architecture was more mosaic, with separated clusters of eosinophilic and paler cells [35]. Distinguishing such tumors from EVT would not be possible without molecular genetic testing. EVT is negative for melanocytic markers (HMB45 or Melan A), which is helpful regarding the differential diagnostic features in relation to AML. The lack of immunoreactivity and the absence of pseudorosettes differentiate EVT from the majority of *TFEB* translocation RCC. For complicated cases, molecular–genetic testing can lead to a definitive correct diagnosis.

The typical cases of SDH deficient RCC are eosinophilic tumors with multiple smaller monotonous intracytoplasmic vacuolization. Such cases have very distinct morphologies and diagnoses of SDH deficient RCC and can be strongly supported by the loss of SDHB expression [20]. In the cases of a strange renal tumor with eosinophilic cytoplasm and features, which might be associated with SDH deficient RCC, at least simple immunohistochemical screening with an antibody against SDHB is recommended. ESC-RCC, LOT, and EVT typically show strong SDHB reactivity.

Interestingly, ESC-RCC, EVT, and LOT may be present concurrently in a single kidney. This is not surprising because of the shared genetic drivers for all three tumors. Such a unique finding was documented in a patient with TSC [19].

**Table 1:** Summary of essential morphological, immunohistochemical, and genetic features.

Entity	Typical Morphology		Immunohistochemical Profile	Molecular Characteristics
ESC-RCC	Cells with abundant eosinophilic cytoplasm, prominent granular cytoplasmic stippling (“eishmanial bodies”)	Combination of solid areas and variably sized macrocystic and microcystic spaces	CK20+ (diffuse or focal), CK7− (or only focally positive), PAX8+, AE1/3+, Vimentin+, CD117−, HMB45−, Melan A−, Cathepsin K+	Recurrent mutually exclusive somatic bi-allelic loss of *TSC1/2*
LOT	Oncocytic cytoplasm, round to oval nuclei, smooth nuclear membrane, focally delicate perinuclear clearing	Solid, compact nested, or focal tubular growth, frequent sharply delineated loose stromal and edematous areas	CK7+ (strong diffuse), CD117−, PAX8+, AE1/3+, CK20−, Vimentin−, HMB45−, Melan A-	Activating *MTOR* mutation/*TSC1* inactivating mutation, recurrent deletion of chromosome 19p, 19q, and 1p, even the disomic pattern
EVT	Large eosinophilic cells, voluminous intracytoplasmic vacuoles, prominent cell membranes, and oval nuclei with enlarged nucleoli	Solid to nested architecture, focally tubulocystic areas	CD117+, CD10+, antimitochondrial antigen antibody+, cathepsin K+, PAX8+, AE1/3+, CK7− (or restricted to rare scattered cells)	Non-overlapping mutations in *mTOR*, *TSC2*, and *TSC1,* deletion of chromosome 1 and 19
ChRCC, eosinophilic variant	Almost purely eosinophilic cells, raisinoid shape of nuclei, and perinuclear clearing	Nested, alveolar, sheet-like architecture	CK7+ (in eosinophilic variant only focally), CD117+, EMA+, CK8+, CK18+, Vimentin-	Most common chromosomal losses: chromosomes 1, 2, 17, 6, 10, 13, 21; no gains of chromosomes [41]
RO	“true oncocytic” cells (cytoplasm stuffed with mitochondria—finely granular appearance of the cytoplasm)	Solid nests in a loose connective stroma	antimitochondrial antigen antibody+, CD117+, CK7−, Vimentin-	Loss of chromosome 1 (whole chromosome or deletion 1p36), 14, or gonosomes (X/Y), 11q13 rearrangement (gene *CCND1*), or normal karyotype [42]
SDH-deficient RCC	Eosinophilic flocculent cytoplasm, numerous intracytoplasmic vacuoles	Solid alveolar architecture	SDHB−, CK7−, CD117−, Vimentin−, PAX8+	Germline mutation of the *SDH* genes (*SDHB/SDHA/SDHC*)
*TFEB* translocation RCC	Two cell populations—large cells with eosinophilic/clear cytoplasm, small eosinophilic cells around basement membrane-like material	Biphasic morphology, rosette-like structures, but wide morphologic spectrum	HMB45+, Melan A+, PAX8+, Cathepsin K+	Translocation with *TFEB* and *MALAT1* gene fusion (most common), other possible partners described (COL21A1, ACTB, EWSR1, CLTC, etc.) [43]
Epithelioid AML	Round to polygonal epithelioid cells, deeply eosinophilic cytoplasm, enlarged vesicular nuclei, prominent nucleoli, focal partial cytoplasmic clearing	Cohesive nests and compartmentalized sheets separated by thin vascular septa/more homogenous growth with diffuse and densely packed sheets	cathepsin K+, HMB45+, Melan A+, AE1/3−, PAX8−	Loss of heterozygosity of *TSC2*, occasional *TFE3* rearrangement [38]

ESC-RCC eosinophilic solid and cystic renal cell carcinoma, LOT low-grade oncocytic tumor, EVT eosinophilic vacuolated tumor, ChRCC chromophobe renal cell carcinoma, RO renal oncocytoma, SDH-deficient RCC succinate dehydrogenase deficient renal cell carcinoma, AML angiomyolipoma, + positive, − negative.

## 6. Conclusions

In addition to AML, several new renal tumors occur in patients with TSC. However, the majority of such neoplasms occur in the sporadic setting.

The recognition of ESC-RCC, LOT, and EVT as novel/emerging renal entities is based on their distinct morphological features and immunohistochemical profiles, while they all share a common molecular–genetic background.

## Data Availability

Data available in a publicly accessible repository.
